# Effect of Laser Irradiation on Emissivity of Flame-Generated Nanooxides

**DOI:** 10.3390/ma14092303

**Published:** 2021-04-29

**Authors:** Silvana De Iuliis, Roberto Dondè, Igor Altman

**Affiliations:** 1 CNR-ICMATE, Institute of Condensed Matter Chemistry and Technologies for Energy, Via R. Cozzi 53, 20125 Milan, Italy; roberto.donde@cnr.it; 2 Combustion Sciences and Propulsion Research Branch, Naval Air Warfare Center Weapons Division, 1 Administration Circle, China Lake, CA 93555, USA

**Keywords:** pyrometry, particulate-generating flame, emissivity, energy gap, electron transitions

## Abstract

The application of pyrometry to retrieve particle temperature in particulate-generating flames strictly requires the knowledge of the spectral behavior of emissivity of light-emitting particles. Normally, this spectral behavior is considered time-independent. The current paper challenges this assumption and explains why the emissivity of oxide nanoparticles formed in flame can change with time. The suggested phenomenon is related to transitions of electrons between the valence and conduction energy bands in oxides that are wide-gap dielectrics. The emissivity change is particularly crucial for the interpretation of fast processes occurring during laser-induced experiments. In the present work, we compare the response of titania particles produced by a flame spray to the laser irradiation at two different excitation wavelengths. The difference in the temporal behavior of the corresponding light emission intensities is attributed to the different mechanisms of electron excitation during the laser pulse. Interband transitions that are possible only in the case of the laser photon energy exceeding the titania energy gap led to the increase of the electron density in the conduction band. Relaxation of those electrons back to the valence band is the origin of the observed emissivity drop after the UV laser irradiation.

## 1. Introduction

Emission properties of flame-generated oxide nanoparticles are a subject of a long-lasting discussion within the combustion community. The interest is caused by a need of spectral emissivity behavior for the interpretation of pyrometry measurements. However, despite decades of research, both the spectral behavior and the absolute value of emissivity remain unknown even for alumina, the most studied oxide. The comprehensive review on alumina [1] reports values that depend on flame conditions and that vary by a couple of orders of magnitude. Another part of the puzzle related to the scatter of the reported alumina emissivity is that alumina is a wide-gap dielectric. Alumina particles should not emit light at all in the visible range, the region corresponding to the material transparency, while a quite strong light emission is usually observed from alumina-generating flames.

The concept suggested in Ref. [2] allows for a possible explanation. The essential condition for the condensation growth of an oxide nanoparticle is a formation of defects within the forbidden band of material and an appearance of non-equilibrium electrons in the conduction band. This is required for dissipating the condensation energy, which is in the order of 5 eV per condensing molecule. Then, the occurrence of defects (which lead to the energy tails in the forbidden band) and non-equilibrium electrons in the conduction band allow for a light emission in the visible range. Since the concentration of defects and non-equilibrium electrons depends on the condensation rate, the inferred nanoparticle emissivity may vary depending on the flame conditions that control this rate.

The justification of this explanation requires multiple steps, which should preferably be done in a system that allows for the most straightforward interpretation of experimental results. This conditioned our choice of the titania-generating flame spray rather than an alumina-producing flame to begin the research. We recently demonstrated the extremely high absorptivity of titania [3] and the coexistence of hot and cold titania nanoparticles in the flame [4]. The latter result is the consequence of the particle condensation growth concept. In this paper, we compare the temporal behavior of the light emitted from the titania flame spray after the irradiation by lasers at two different excitation wavelengths, namely IR (1064 nm) and UV (266 nm).

The idea of this comparison is based on a relationship between the titania energy gap and the laser photon energy. The photon energy of the UV laser (4.66 eV) exceeds the energy gap of titania (3.2 eV), while that of the IR laser (1.17 eV) is less than the energy gap. The difference between the laser photon energies leads to different electron excitation mechanisms under laser irradiation. In the case of the UV laser, interband electron excitations are possible, i.e., the number of electrons in the conduction band increases during the laser pulse. This excess electron concentration decays after the laser pulse due to electron transitions back to the valence band. Then, the contribution of these excess electrons to the particle emissivity also decreases after the UV laser pulse. On the contrary, in the case of IR laser irradiation, which does not generate excess electrons in the conduction band, the particle emissivity does not change with time. The spectral behavior of emissivities can be also different. As far as the IR laser irradiation does not generate excess electrons, in this case the emissivity of the particle, whose structure is highly disordered, can be described by the Urbach law [3]. The excess electrons that are excited by the UV irradiation contribute to the additional (metal-like) term in the particle emissivity. Then, the resulting emissivity after the UV laser pulse is expected to be a weaker function of the wavelength compared to that after the IR laser pulse. We apply this concept interpreting the temporal difference in the laser-induced emission from flame-generated titania nanoparticles under two different excitation wavelengths.

## 2. Processing of Flame Light Emission Spectra

We use the spectra processing procedure described in detail in Ref. [4]. The light intensity, *I_λ_*, that is emitted by nanoparticles can be expressed as,

(1) $$I_{\lambda }=Af_{V}q\left(\lambda \right)W\left(T,\lambda \right)\;,$$

where *W* is the Wien function, which depends on the temperature, *T*, and the wavelength, *λ.* The constant *A* accounts for the probe volume and *f_V_* is the volume fraction of particles that emit light; *q*(*λ*) is the explicit function of the complex refractive index of the particle material. In the Rayleigh limit *q*(*λ*) is proportional to the spectroscopic function *E*(*m*) as *q*(*λ*) ~ *E*(*m*)/*λ*, where *m* = *n* − i*k* is the complex refractive index, which depends on the wavelength. It should be noted that in Equation (1) the Wien function is not the Planck function approximation, but it is the consistent term describing the flame radiation emitted by particles in steady-state (not equilibrium) conditions [4,5].

Re-writing Equation (1) as

(2) $$\ln\left(I_{\lambda }\lambda^{5}\right)=\ln\left(A\right)+\ln\left(f_{V}\right)+\ln\left[q\left(\lambda \right)\right]-\frac{C_{2}}{\lambda T}\;,$$

where *C*_2_ = 14,388 µm·K is the second radiation constant commonly used in the Planck law, we can see that the slope, *K*, of the linearly interpolated Wien plot (ln(*I_λ_·λ*^5^) plotted as a function of the reciprocal wavelength) gives the gray body radiation temperature, *T^g^*, as

(3) $$T^{g}=-\frac{C_{2}}{K}\;.$$

Note that the gray body temperature is defined as that at *q*(*λ*) = constant. The particle volume fraction determines the intercept of the extrapolated straight line with *Y*-axis. For two measurements with the same probe volume, the intercept change is $\ln\left(f_{V,2}/f_{V,1}\right)$, if the function *q*(*λ*) is the same for both systems. Then, in order to infer the relative volume fraction, $f_{V,2}/f_{V,1}$, the change of intercept can be used.

If the radiation intensities are measured at given wavelengths, the standard least squares regression procedure can be utilized for processing the data obtained. In particular, *Y* variables are defined as the experimental $\ln\left(I_{\lambda }\lambda^{5}\right)$ values, *X* variables are defined as the reciprocal wavelengths, *1/λ* and the regression line is sought in the form *Y* = *a + bX*. Then, the gray body temperature is obtained as *T^g^* = −14,388/*b*. For two measurements in systems of the same probe volume, the relative volume fraction is obtained as $f_{V,2}/f_{V,1}$= exp(*a_2_* − *a_1_*).

We processed all recorded light emission spectra as described above to get the gray body temperature and the relative volume fraction of the irradiated flame after the laser pulse. It should be emphasized that the gray body temperature defined by Equation (3) is a characteristic of the emitted radiation, but it is not an actual system temperature. This notion is important for understanding the seemingly puzzling behavior of the obtained temperature we further discuss.

## 3. Experimental

**Figure 1:**
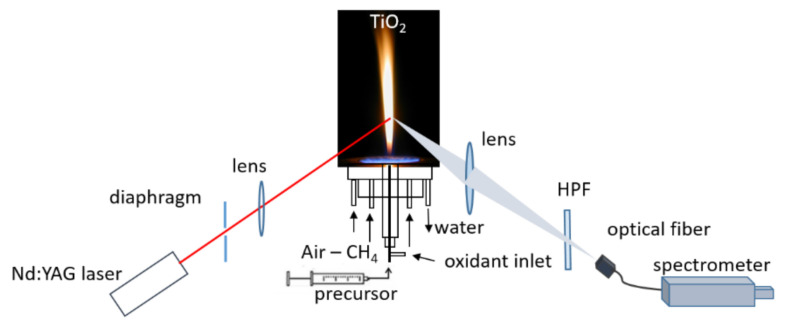
Experimental setup of light emission measurements as reported in Refs. [3,4,6].

The light emission measurements were carried out using the experimental setup presented in Figure 1, see also Refs. [3,4,6]. The fundamental frequency beam (IR, 1064 nm) of a pulsed (5 Hz, 7ns pulse duration) Nd:YAG laser (Big Sky CFR 400, Quantel USA, Bozeman, MT, USA) and the fourth harmonic (266 nm) of a pulsed (5 Hz, 5ns pulse duration) Nd:YAG laser (SYL Nd:YAG, Quanta System S.p.A., Samarate, Italy) were used. In particular, a diaphragm (Ø = 4 mm) was used in order to select a portion of the laser beam that was sent into the flame by means of an 1190 mm lens. A lens (focal length f = 22.5 cm) collected and focused the light emitted by the nanoparticles on an optical fiber being connected to the entrance slit of a spectrograph (Shamrock 303i) that was coupled with an ICCD camera (iSTAR 334T, Andor Technology, Belfast, UK). A spatial resolution of the nanoparticle radiation measurements was estimated to be 3 mm, which took into account the optical fiber diameter of 3 mm and the magnification of the collection optics of 1:1.

A high-pass filter (305 nm, CVI Melles Griot, Albuquerque NM, USA, HPF in the Figure 1) was positioned in front of the optical fiber. It was used in order to remove the second order diffraction of the spectrometer. The spectral light emission was collected with a 150 grooves/mm grating, allowing for a 0.28 nm spectral resolution. The spectral response of the optical receiving optics and detection was evaluated and corrected with the help of a calibrated tungsten lamp (200 W, Oriel 63,355, Newport Corporation, Stantford, CT, USA), whose radiation was measured in the same geometry as that emitted by nanoparticles.

Light emission measurements were carried out on titania nanoparticles. An in-house developed Flame Spray Pyrolysis (FSP) apparatus that allowed for these nanoparticles’ production is widely described elsewhere [6]. We here briefly summarize only few details of the experimental conditions and the flame analyzed in the current work. In FSP, which is essentially an oxygen-assisted spray apparatus, the precursor can be injected coaxially with the pilot flame. For the pilot flame, we used a laboratory-built water-cooled lamella burner, which produced a sustaining premixed lean flame with an equivalence ratio (methane/air) of 0.8. A homemade stainless steel gas-assisted spray injector was utilized to create the spray. This injector consisted of a capillary with 0.3 mm inner diameter and 0.8 mm outer diameter that was inserted in a 1.2 mm diameter gas nozzle. An oxygen stream was utilized as a nebulizing gas that flowed the precursor solution through the capillary. The flame conditions (i.e., gas velocity and, correspondingly, residence time as well as oxygen/fuel ratio affecting the temperature field) could be changed by varying flow rates.

As a liquid precursor, we used titanium tetraisopropoxide (97% purity, Sigma-Aldrich, St. Louis, MO, USA) dissolved in ethanol (0.5 M). In the experiment, the precursor solution was injected through the spray nozzle at 4 mL/min feed rate using a syringe pump and the flow rate of oxygen stream of 5 Nl/min was used for nebulizing. It resulted in a synthesis diffusion flame of about 8 cm height. The obtained flame exhibited a very strong whitish light emission. By means of vacuum pump system, titania nanoparticles were collected on a glass fiber filter (150 mm diameter, Whatman, Maidstone, UK, Grade *G*F/A Glass Microfiber filter) downstream of the reactor allowing for further ex-situ characterizations. Under the above experimental conditions, the nanoparticle diameter measured by the transmission electron microscope analysis was about 20 nm.

In the current paper, we studied nanoparticle light emission intensities that were collected from the non-irradiated flame and the flame irradiated by the laser at different delay times (100–800 ns) after the laser pulse (IR and UV), and compared the temporal behavior of the gray body temperature and the relative volume fraction inferred according to the procedure described in the previous section. Measurements were performed at a 2 cm height above the burner (HAB). The delay time range was chosen as it was expected to be long enough for the noticeable light emission spectra evolution after the laser shot, and it was much shorter than the residence time of a nanoparticle in the probe volume. The latter was estimated based on the utilized flow rates to be in the order of tens of microseconds. In all measurements, signals were alternately collected with and without the laser, respectively, as described in Ref. [6]. This allowed one to check the effect of the laser irradiation on the nanoparticle light emission, i.e., to discriminate between the emission of nanoparticles in the laser-irradiated flame from that in the non-irradiated flame. A total of 100 single shots were collected to acquire data at each delay time.

## 4. Results and Discussion

In order to investigate the different responses of titania nanoparticles under IR and UV excitation, substantially different laser fluences are required to get a comparable change in the flame emission intensity, with the titania absorption properties in the UV spectra range substantially higher than in the IR. For this reason, the fluence of the UV laser was chosen at 80 mJ/cm^2^, which was used in our previous work [6], while for the IR laser fluence the highest value of 562 mJ/cm^2^ was used. The latter allowed for the flame emission intensity change at the level comparable to that in the UV laser experiment.

**Figure 2:**
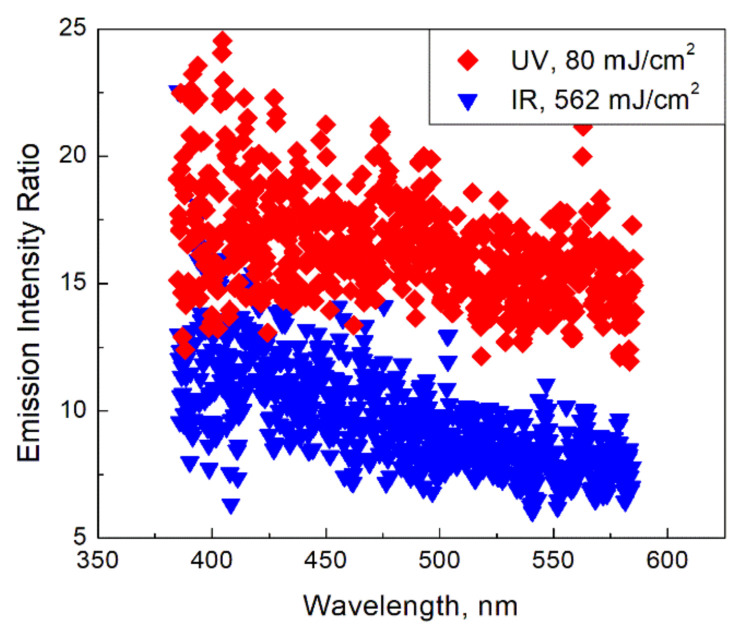
Ratio of emission intensities of the irradiated (at 100 ns delay time after the laser pulse) and non-irradiated flame for lasers of different wavelengths.

In Figure 2 the ratio of the emission intensities of irradiated and non-irradiated flames are shown at the delay time of 100 ns for the two excitation wavelengths. The effect of the UV irradiation on the flame emission intensity looks noticeably stronger than that of the IR irradiation.

**Figure 3:**
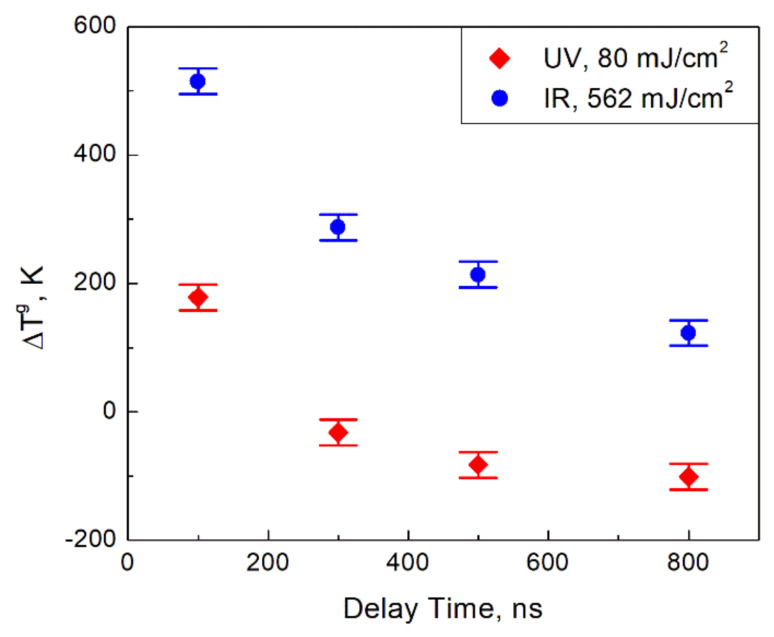
The change of the gray body temperature at different delay times after the laser pulse. Uncertainties are estimated based on errors of the fitting procedure returning the regression coefficients.

**Figure 4:**
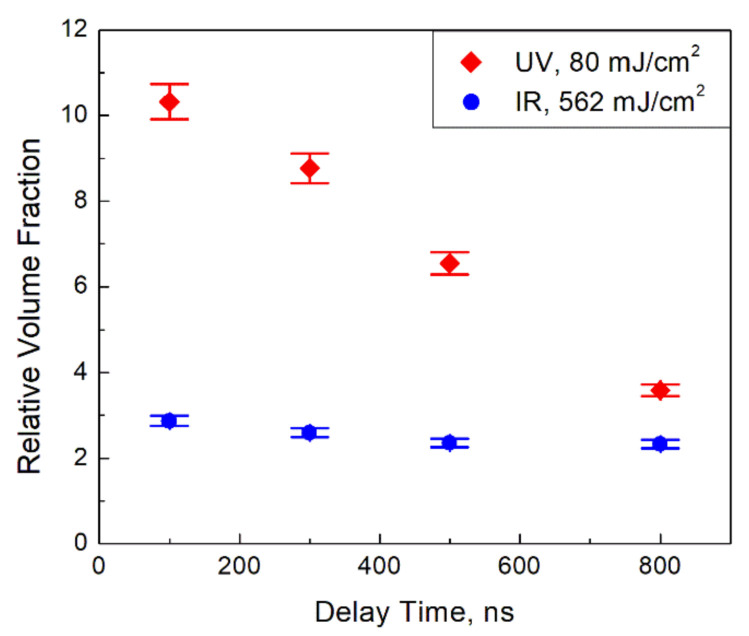
The relative volume fraction after the laser irradiation at different delay times after the laser pulse. Uncertainties are estimated based on errors of the fitting procedure returning the regression coefficients.

Following the procedure detailed in Section 2, we inferred the gray body temperature of the irradiated and non-irradiated flames and the relative volume fraction at different delay times. In Figure 3, the change of the gray body temperature, which is defined as the difference between the gray body temperature of the laser-irradiated flame at a given delay time and the gray body temperature of the non-irradiated flame, is presented at different delay times after the laser pulse. In Figure 4, the relative volume fraction, which we define as the ratio of the volume fraction of the laser-irradiated flame at a given delay time and the volume fraction of the non-irradiated flame, is shown. Uncertainties of the reported values are based on errors of the fitting procedure returning the regression coefficients *a* and *b*. The typical temperature error originated from the fitting uncertainty of the coefficient *b* was estimated to be about 10 K, so the total error of the temperature difference is about 20 K. The fitting error of the intercept, i.e., the coefficient *a*, was about 0.02 which corresponds to the relative error of 2%. Then, taking into account the definition of the relative volume fraction, we estimated its relative error as 4%.

As one can see in Figure 3, the irradiation by the IR laser leads to a higher change of the gray body temperature compared to the UV laser, which looks contradictory with the laser effect on the emission intensity presented in Figure 2. Indeed, a higher change of temperature is expected to lead to a higher increase of the emission intensity, which seems not to be the case. As it was mentioned above, a possible explanation of the paradox is that the change of the gray body temperature has nothing to do with the change of the actual temperature, which should be likely higher for the flame irradiated by the UV laser. We will return to that relationship between the gray body and actual temperatures later.

The different character of the temporal behavior of the relative volume fraction seen in Figure 4 also deserves mentioning. As it was demonstrated in Ref. [4], this value is just a parameter of the data processing we utilize. Then, the different temporal behavior inferred is evidence of some difference of particle properties depending on the wavelength of irradiation. Note that the increase of the relative volume fraction of 2–3 times after the IR pulse, which was analyzed in Ref. [4], is due to the coexistence of the particles of different temperatures (hot and cold) in the flame. The cold particles, invisible in the non-irradiated flame, become visible in light emission after the laser pulse. Then, the relative volume fraction remains nearly constant after the IR laser pulse.

Besides the heating, the UV irradiation (whose photon energy exceeds the titania energy gap) can change the electrical properties of the affected material, i.e., excite electrons from the valence band to the conduction band. The latter process is impossible in the case of the IR irradiation with the photon energy less than the energy gap. The interband transitions induced by the UV laser lead to the increase of the absolute value of the particle emissivity, *q*(*λ*) in Equation (1). This increase is interpreted as the increase of the volume fraction, since our data processing does not formally take into account the *q*(*λ*) change. The relaxation of the excited electrons back to the valence band leads to the decrease of *q*(*λ*) from its elevated value after the laser pulse, which is interpreted as the drop of the relative volume fraction seen in Figure 4. The characteristic time of the electron relaxation in the order of hundreds of nanoseconds required for the drop explanation looks reasonable.

The occurrence of the excited electrons in the conduction band can also explain the paradox with the gray body temperatures mentioned above. By definition, the gray body temperature is a parameter describing the spectrum of light emission based on assumptions that there is no spectral dependence of the emissivity of an emitting body. As one can understand, being just a fitting parameter, the gray body temperature has nothing to do with the actual body temperature in general. The relationship between these two temperatures depends on the character of the emissivity of the light emitter. If the emissivity is a strong function of the wavelength, the difference between the gray body and actual temperature is large. Otherwise, in the case of a weak spectral dependence, the gray body and actual temperatures may be close to each other.

As was discussed in Ref. [3], for flame-generated oxides, whose emissivity is determined by transition between tail energy states, the Urbach law (see Ref. [7] and references within) is a good approximation,

(4) $$q\left(\lambda \right)\propto B\;\exp\left(\frac{E}{E_{U}}\right)=B\;\exp\left(\frac{C_{2}}{\lambda \left(\frac{E_{U}}{k_{B}}\right)}\right)\;,$$

where *B* is the pre-factor and *k_B_* is the Boltzmann constant. The Urbach energy, *E_U_*, depending on the defect concentration in the forbidden band may be quite large for the titania particles generated in the flame. Equations (2)–(4) lead to the explicit relationship between the gray body temperature, *T^g^* and the actual temperature, *T_a_*, as

(5) $$T_{a}=\frac{T^{g}}{1+k_{B}T^{g}/E_{U}}\;,\;$$

(6) $$T^{g}=\frac{T_{a}}{1-k_{B}T_{a}/E_{U}}\;.\;$$

Thus, the actual temperature is always lower than the gray body temperature inferred by fitting the light spectra emitted by flame-generated nanooxides. The greater the Urbach energy (i.e., the weaker spectral dependence of the emissivity) the smaller the difference between these temperatures.

The interband electron excitations induced by the UV laser enable an additional mechanism of the light emission, which is related to the occurrence of the electrons in the conduction band and their intraband (within the conduction band) transitions. This mechanism is the same as that providing light emission from metals. Similarly to metals, its contribution to the overall particle emissivity is expected to be weakly dependent on the wavelength. Obviously, this additional contribution does not appear after the IR laser irradiation, whose photon energy is not high enough to excite electrons from the valence band to the conduction band. Then, the resulting particle emissivity of the nanooxide particle after the UV irradiation is a weaker function of the wavelength compared to that described by Equation (4). Therefore, the difference between the gray body and actual temperatures is smaller for nanoparticles exposed to the UV laser compared to those exposed to the IR laser. Correspondingly, at the same actual temperatures, the UV-irradiated particles have a lower gray body temperature compared to the IR-irradiated particles. This finally explains why the change of the gray body temperature is stronger after the IR laser pulse compared to that after the UV laser pulse, as it is seen in Figure 3. Moreover, at any delay time, the emissivity of the particles irradiated by the UV laser is a weaker function of the wavelength compared to that in the non-irradiated flame. Then, at some delay time, the gray body temperature of the UV-irradiated particles can become lower than the gray body temperature of the particles in the non-irradiated flame (which is seen in Figure 3 at a delay time exceeding 200 ns), although the relationship between corresponding actual temperatures is obviously opposite.

The results here reported can have a significant impact on pyrometry measurements, which is considered a powerful diagnostic tool for the characterization of the particulate-generating flames. The method proposed can be also applied for determining temperatures in high-pressure high-temperature studies [8]. We have seen that due to the occurrence of different mechanisms under laser irradiation with different excitation energies, different spectral behaviors of emissivity should be considered for a proper evaluation of the effective particle temperature. In the case of the flame-generated particles, several energy ranges are involved due to inception/coagulation/condensation mechanisms responsible for the particle growth. These may significantly affect emission properties of the studied particles. Consequently, care has to be taken for the emission intensity processing in order to retrieve a reliable particle temperature.

## 5. Concluding Remarks

The comparison of the temporal behavior of the spectra emitted by titania particles generated by a flame spray after the laser irradiation of different wavelengths carried out in the current paper revealed a noticeable difference in the effect of the laser photon energy on the character of the evolution of the flame characteristics inferred from the light emission spectra. The anomalous behavior found in case of the UV laser is ascribed to the possibility of the laser-induced electron excitation from the valence band to the conduction band and to processes following this excitation. The suggested mechanism looks important for optical diagnostics in systems with energy ranges comparable to the energy gap. In particular, the time dependence of the particle emissivity needs to be taken into account for further comprehension of the pyrometry interpretation in the particulate-generating flames.

## Data Availability

The data presented in this study are available on request from the corresponding author.
